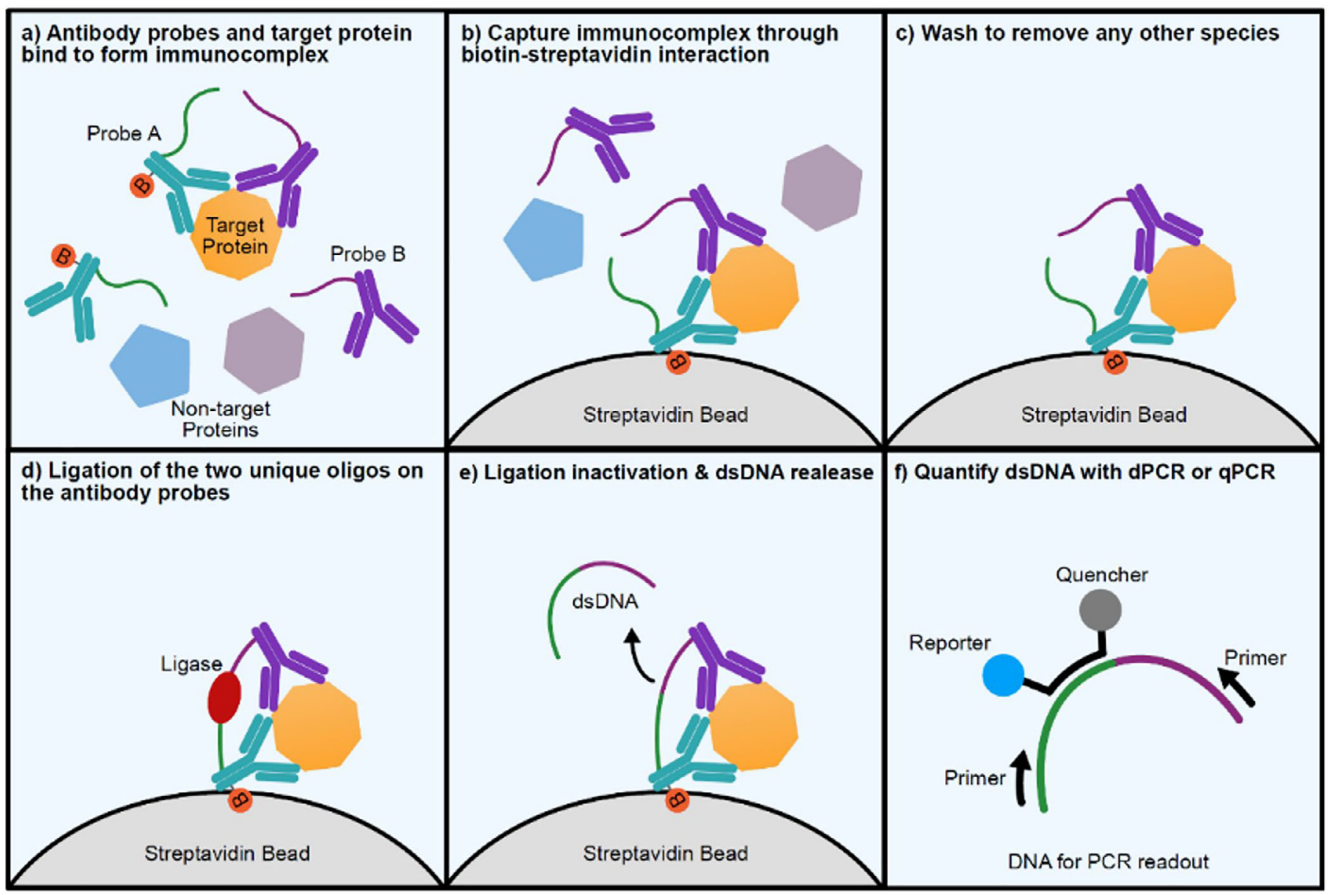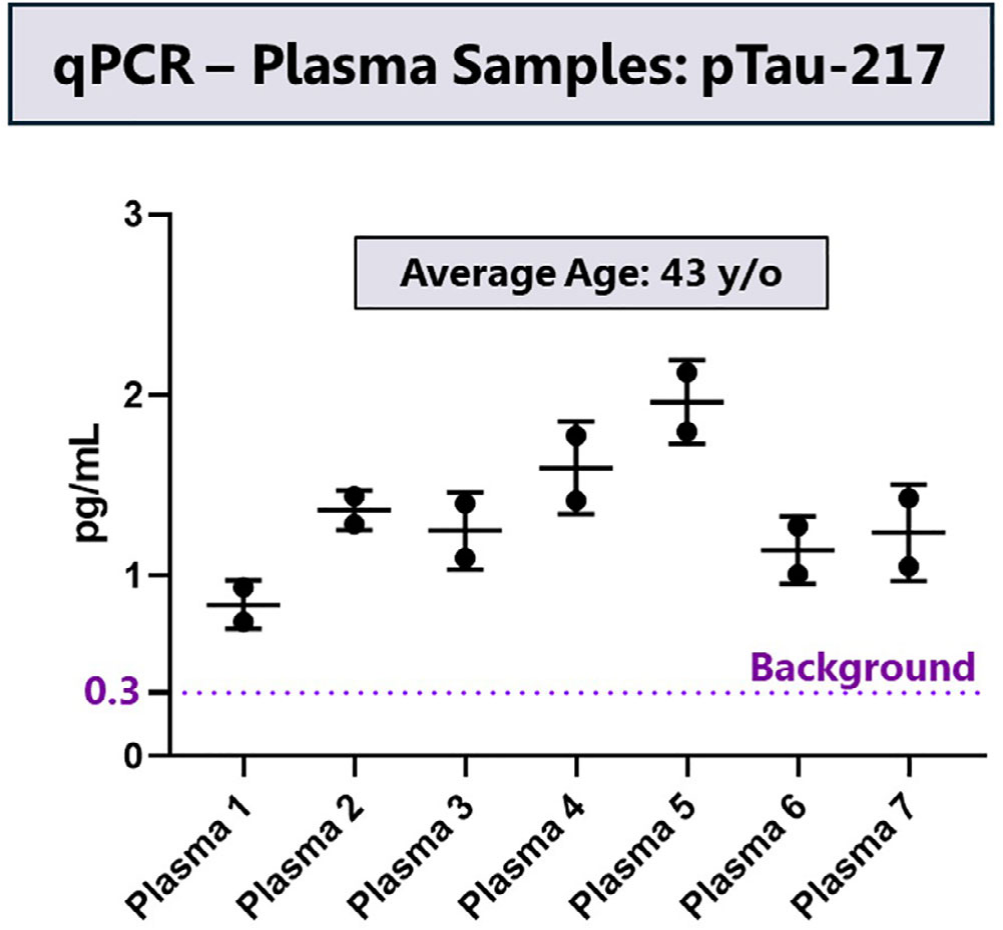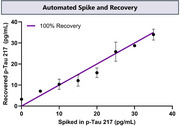# Fully automated, sample‐to‐answer platform for the quantitation of blood‐based biomarkers for early detection of Alzheimer's disease

**DOI:** 10.1002/alz70856_096631

**Published:** 2025-12-24

**Authors:** Robert Lin, Hien Nguyen, Jason Wan

**Affiliations:** ^1^ Taudia, PALO ALTO, CA, USA

## Abstract

**Background:**

Blood‐based biomarkers (BBMs) have key advantages including more cost efficient, easier to access, and less invasive than traditional diagnostic methods such as Positron Emission Tomography (PET) and cerebrospinal fluid (CSF) testing. For Alzheimer's Disease (AD), pTau‐217 has emerged as the most promising biomarker due to its high clinical relevance and specificity. Despite advancements in biomarker discovery, no sample‐to‐answer, multiplexing diagnostic platform focused on neurodegeneration has been introduced to the market. To address this gap, we present here the first sample‐to‐answer multiplexing diagnostic platform specifically designed for AD.

**Method:**

We combine a novel antibody modification approach with a unique background signal minimizing bead‐based assay to improve both the sensitivity and specificity of traditional proximity ligation assay (PLA). Unlike conventional PLA, where each antibody is conjugated to an oligonucleotide, one of our antibody pairs is conjugated to an oligonucleotide and a capture moiety using our patented dual conjugation strategy. This dual‐conjugation PLA (dcPLA) approach allows extraction to be completed with a single wash step (Figure 1), significantly reducing workflow complexity, enhancing consistency, and enabling direct integration with a sample‐to‐answer system. The platform technology can also be easily applied to other antibodies for menu expansion.

**Results:**

We used dcPLA to detect and quantify endogenous pTau‐217 levels in seven healthy plasma samples acquired from a commercial source with high consistency and high signal‐to‐noise ratio. (Figure 2)

Additionally, we demonstrated a fully automated sample‐to‐answer diagnostics platform using the novel dcPLA with a single, streamlined workflow. The platform can process up to 48 samples in a single batch, in around five hours and requires no hands‐on time beyond the initial loading of samples and reagents. Here we demonstrate an automated spike and recovery experiment show close to 100% recovery with 3X diluted plasma. (Figure 3)

**Conclusion:**

Here, we present the first AD‐focused diagnostic platform combining an FDA‐approved Class 1 sample‐to‐answer system with the novel dcPLA technology. By enabling minimally invasive and scalable testing, the platform has the potential to assist drug developers recruiting the right patients for their clinical trials, paving the way toward early detection, longitudinal monitoring, and personalized treatment strategies to improve AD patient outcome worldwide.